# Complex Chromothripsis-like Features in Plasma Cell Myeloma: A Case Report and Review of the Literature

**DOI:** 10.3390/diagnostics16091280

**Published:** 2026-04-24

**Authors:** Jaymie Oentoro, Sonia Yu, Kevin A. Murgas, Jacob Rocha, Tahmeena Ahmed, Carlos A. Tirado

**Affiliations:** 1 Department of Pathology, Stony Brook University Hospital, Stony Brook, NY 11794, USA; 2 Division of Hematology and Oncology, Department of Medicine, Stony Brook University Hospital, Stony Brook, NY 11794, USA; 3 Medical Scientist Training Program, Renaissance School of Medicine, Stony Brook University, Stony Brook, NY 11794, USA

**Keywords:** plasma cell myeloma, multiple myeloma, chromosomal instability, chromothripsis

## Abstract

**Background** **and Clinical Significance:** Chromothripsis represents a catastrophic genomic event in plasma cell myeloma (PCM) associated with poor prognosis. We report a case of newly diagnosed PCM with complex cytogenetic abnormalities indicative of genomic instability. **Case Presentation:** A 67-year-old man presented with acute dyspnea and was found to have severe acute kidney injury, anemia, hypercalcemia, and IgG lambda monoclonal gammopathy. Bone marrow biopsy revealed plasma cell infiltration. Comprehensive FISH analysis demonstrated a complex pattern with gain of 1q, monosomy 13, and multiple numeric and structural abnormalities affecting chromosomes 5, 9, and 15, suggestive of a chromothripsis-like pattern. Despite requiring hemodialysis, the patient achieved complete renal recovery and >99% reduction in serum-free light chains after one cycle of CyBorD plus daratumumab, which was continued for four cycles. Follow-up bone marrow evaluation at three months confirmed complete histologic, flow cytometric, and cytogenetic remission, allowing for preparation for autologous stem cell transplantation. **Conclusions:** This case demonstrates that exceptional clinical responses can be achieved in high-risk disease with contemporary quadruplet regimens. While the long-term durability of such responses in genomically unstable cases remains uncertain, this case highlights the importance of comprehensive cytogenetic characterization to identify and monitor genomic instability in PCM.

## 1. Introduction

### 1.1. Plasma Cell Myeloma: Overview and Pathogenesis

Plasma cell myeloma (PCM), also known as multiple myeloma, is an incurable plasma cell malignancy characterized by clonal proliferation of plasma cells in the bone marrow. The disease develops through a multistep process beginning with initiating events, often hyperdiploidy or translocations involving immunoglobulin gene loci, which can occur in precursor conditions such as monoclonal gammopathy of undetermined significance (MGUS) and smoldering myeloma [1,2]. These primary events are followed by acquisition of secondary genetic alterations, including copy number abnormalities, secondary translocations, and somatic mutations that drive progression to symptomatic disease [1,2,3,4].

### 1.2. Chromosomal Instability and Chromothripsis: Definition and Mechanisms

Chromosomal instability is a hallmark feature of PCM, contributing to the heterogeneous genomic landscape that characterizes this malignancy; genomic complexity typically escalates as disease progresses from MGUS to newly diagnosed and relapsed/refractory disease [5,6]. In this context, chromothripsis represents a catastrophic form of genome instability first described by Stephens et al. in 2011 [7]. This phenomenon is characterized by fragmentation of one or more chromosomes, often sequestered into micronuclei after defective mitosis, resulting in tens to hundreds of locally clustered DNA breaks [8,9,10,11]. Cells may survive this catastrophic event through robust engagement of DNA repair pathways, primarily non-homologous end joining and replication-associated processes, which allow for rapid rejoining of chromosome fragments despite extensive rearrangements [12,13,14]. The resulting genomic hallmarks of chromothripsis include oscillations between two copy number states, localized clustering of rearrangements, and apparently random reassembly of chromosomal fragments [12,14,15]. This fragmentation and error-prone repair can lead to further loss of tumor suppressor function, creation of fusion genes, and activation of oncogenes [16].

### 1.3. Chromothripsis in Myeloma: Prevalence and Detection

The reported frequency of chromothripsis in PCM is highly dependent on detection sensitivity. Early studies using SNP array data identified chromothripsis in only 1.3% of newly diagnosed patients, while more recent studies using high-resolution microarrays have reported intermediate frequencies of approximately 10% [17,18]. Whole genome sequencing studies, often considered the gold standard for identifying complex genomic events, have established a prevalence of chromothripsis in 20–30% of newly diagnosed PCM [19,20]. Emerging technologies, including optical genome mapping and copy number signatures, offer promising tools to enhance sensitivity in the clinical diagnostic setting, though standardization of criteria remains an ongoing challenge [21,22].

### 1.4. Clinical and Prognostic Significance of Chromothripsis in Myeloma

Chromothripsis has emerged as an independent adverse prognostic factor in PCM consistently associated with inferior progression-free and overall survival [17,20]. Unlike the gradual accumulation of genetic hits seen in standard-risk disease, the catastrophic nature of chromothripsis often drives rapid development of therapeutic resistance and early relapse [20,22]. Notably, recent data indicate that chromothripsis is a strong independent predictor of poor outcomes even when adjusting for standard high-risk variables [18,22,23].

Current risk stratification systems define specific high-risk cytogenetic abnormalities. While the Revised International Staging System (R-ISS) originally specified del(17p), t(4;14), and t(14;16), the Mayo Stratification for Myeloma and Risk-adapted Therapy (mSMART) guidelines additionally included *TP53* mutation, t(14;20), 1q gain/amplification, and del(1p) [24,25,26]. This broadened definition was recently validated by the 2025 International Myeloma Society and International Myeloma Working Group Consensus Genomic Staging (IMS-IMWG CGS), which formally classified 1q21 gain in combination with other cytogenetic abnormalities as high-risk [27,28]. Although chromothripsis is not yet a standalone criterion in these algorithms, its strong association with complex karyotypes and “double-hit” genetic profiles suggests it represents a distinct biological category that warrants consideration in comprehensive risk assessment [17,22,23].

Herein, we present a case of newly diagnosed PCM with comprehensive cytogenetic characterization revealing a high-risk complex karyotype with features suggestive of a chromothripsis-like pattern. Notably, the patient achieved remarkable clinical response to quadruplet therapy, highlighting both the prognostic importance of detailed cytogenetic analysis and the potential to effectively manage high-risk disease with contemporary treatment regimens.

## 2. Case Presentation

### 2.1. Clinical Presentation

A 67-year-old man with no known medical history presented with 3 days of progressive dyspnea. Physical examination revealed mild hypoxemia with oxygen saturation of 92% on room air. Initial laboratory evaluation demonstrated severe acute kidney injury (creatinine 10.9 mg/dL), anemia (hemoglobin 8.0 g/dL), hyperkalemia (potassium 5.4 mmol/L), hypercalcemia (corrected calcium 11.5 mg/dL), and markedly elevated NT-proBNP of 28,000 pg/mL. Serum lactate dehydrogenase was 289 IU/L. A significant protein gap was noted with total protein of 11.8 g/dL and albumin of 2.6 g/dL. Urinalysis revealed proteinuria of 100 mg/dL. Computed tomography of the chest demonstrated a small pleural effusion and pulmonary edema. Given oliguria in the setting of progressive renal dysfunction, electrolyte derangement, metabolic acidosis, and hypervolemia, hemodialysis was initiated.

### 2.2. Laboratory and Pathologic Findings

**Figure 1:**
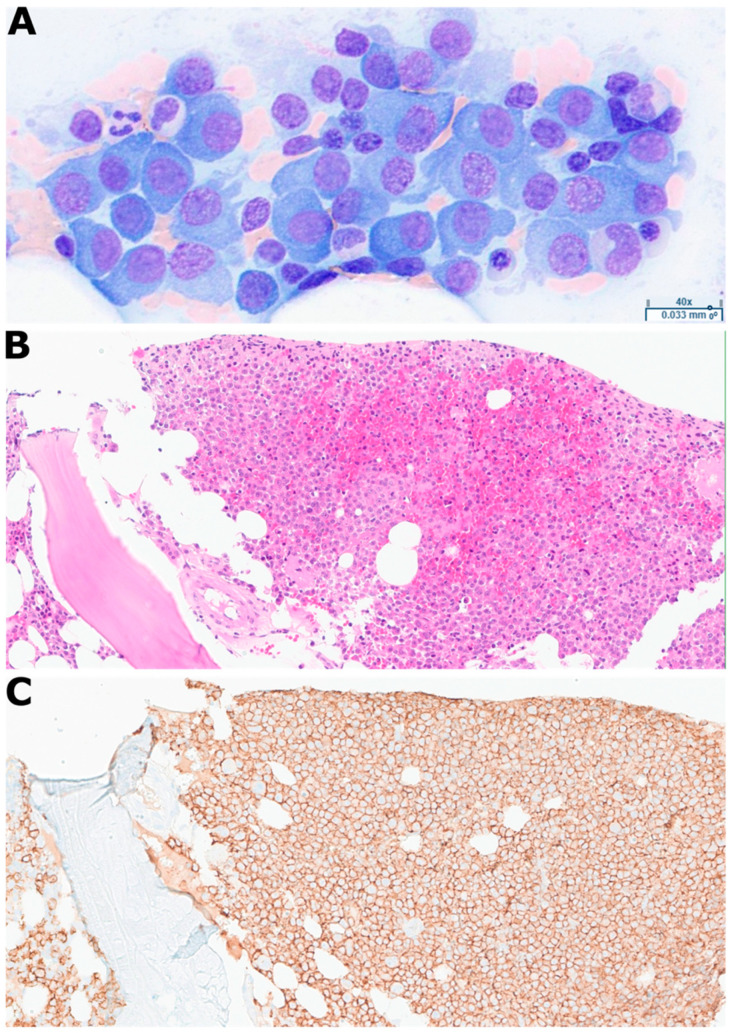
**Histological characterization of genomically complex plasma cell myeloma case.** (**A**): Wright–Giemsa stain of bone marrow touch-prep showing hypercellular sheets of plasma cells with abnormal plasmablastic and multinucleated forms. (**B**): Hematoxylin and eosin stain of bone marrow core biopsy showing sheets of plasma cells. (**C**): CD138 immunohistochemical stain of core biopsy (adjacent section to panel **B**) showing >95% of total cells. Together, these findings confirm near-total replacement of marrow architecture by an aggressive, morphologically abnormal plasma cell neoplasm.

Serum and urine immunofixation both demonstrated IgG lambda monoclonal restriction with serum-free lambda light chains of 3850 mg/L and serum IgG of 7370 mg/dL. Given the positive paraprotein studies in the setting of severe renal dysfunction, an urgent bone marrow biopsy was performed on hospital day 3, which revealed a hypercellular marrow with sheets of plasma cells comprising 65% marrow cellularity. The aspirate and touch-prep were hypercellular and showed sheets of plasma cells, with abnormal plasmablastic and multinucleated forms (Figure 1A). The core biopsy showed sheets of plasma cells highlighted by CD138 immunohistochemical stain (>95% of total cells) (Figure 1B,C).

Kidney biopsy performed on hospital day 6 confirmed lambda light chain cast nephropathy. Cardiac magnetic resonance imaging and abdominal fat pad biopsy showed no evidence of amyloidosis.

### 2.3. Cytogenetic Methodology

Comprehensive cytogenetic evaluation was performed using fluorescence in situ hybridization (FISH) on CD138-enriched plasma cells. Multiple probe sets were utilized, including the AneuVysion MulticolorDNA Probe Kit (Vysis CEP18/X/Y-alpha satellite) (Abbott, Des Plaines, IL, USA), the *ABL2*(1q25.2) BA FISH probe (Empire Genomics, Williamsville, NY, USA), the LSI 1p36/1q25, the LSI D5S23,D5S721/CEP 9/CEP 15, the LSI 13/*RB1*(13q14), the LSI *RUNX1*/*RUNX1T1*(8q21.3,21q22), the LSI *MYC* DC BAR (8q24), and the LSI *SNRPN*/CEP15/*PML* probe panels (Abbott, Des Plaines, IL, USA).

### 2.4. Cytogenetic Findings

**Figure 2:**
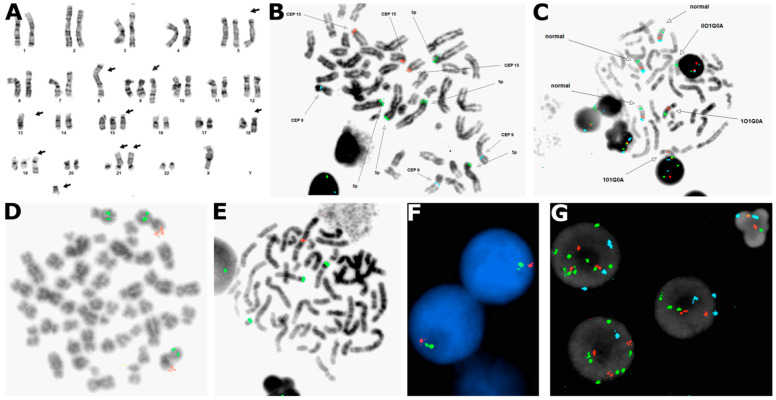
**Chromothripsis-like genomic rearrangements in a plasma cell myeloma case.** (**A**): Representative karyotype demonstrating numerous complex chromosomal events. (**B**): Metaphase chr5/9/15 FISH panel with D5S23,D5S721/5p15.2 probe in green, CEP9/9p11-q11 probe in aqua, and CEP15/15p11.1-q11.1 probe in orange demonstrating copy gains in each target. (**C**): Metaphase Prader–Willi three-color FISH panel demonstrating multiple copies of chromosome 15 with variable partial aneuploidy. (**D**): Metaphase CEP1 FISH panel, with 1p36 probe in orange and 1q25 probe in green, demonstrating trisomy 1 with partial loss of 1p in one copy. (**E**): Metaphase t(8;21) FISH fusion panel, with RUNX1/8q21.3 probe in green and RUNX1T1/21q22 probe in orange, demonstrating trisomy 8 and monosomy 21. (**F**): Interphase Rb1 FISH panel, with Rb1/13q14 probe in red and 13qter control probe in green, demonstrating unisomy 13. (**G**): Interphase SNRPN/CEP 15/PML FISH panel, with SNRPN/15q11.2 in orange, D15Z1/15q11-13 in aqua, and PML/15q24 in green, demonstrating complex aneuploidy of chr15. Collectively, this constellation of concurrent structural rearrangements, localized chromosomal shattering, particularly involving chromosome 15, and multiple high-risk copy number variations visually illustrates the severe genomic instability characteristic of a chromothripsis-like event. Probes used: LSI 1p36/1q25, LSI D5S23,D5S721/CEP 9/CEP 15, LSI 13/RB1(13q14), LSI RUNX1/RUNX1T1(8q21.3,21q22), and LSI SNRPN/CEP 15/PML probe panels (Abbott, Des Plaines, IL, USA).

Metaphase and interphase FISH analysis, alongside conventional karyotyping (Figure 2A), demonstrated a complex cytogenetic pattern with multiple numerical abnormalities suggestive of a chromothripsis-like pattern. Targeted metaphase analysis confirmed high-risk abnormalities and extensive instability. We observed a gain of 1q25 in 50.5% (101/200) of nuclei, a known adverse prognostic marker (Figure 2B). Chromosome 15 demonstrated significant instability, with metaphase spreads revealing variable partial aneuploidy involving complex intra-chromosomal amplification (Figure 2C). Structural rearrangements involving chromosomes 8 and 21 were also characterized; specifically, one copy of *RUNX1T1* (8q21.3) and three copies of *RUNX1* (21q22) were seen in 40% (80/200) of nuclei, consistent with monosomy 8 and trisomy 21 (Figure 2D). Additionally, multi-probe panels highlighted numerical instability, including three to five copies of 5p15.2 in 55% (110/200) of nuclei, three copies of chromosome 9 in 22% (44/200) of nuclei, and three copies of chromosome 15 in 33% (66/200) of nuclei (Figure 2E). Loss of the Y chromosome was also observed in 31% (62/200) of nuclei. Interphase analysis further defined the clonal architecture, confirming monosomy 13 (Figure 2F). Finally, the complex nature of the chromosome 15 rearrangements was resolved through interphase probing, which showed three copies of CEP 15, five copies of *SNRPN* (15q11.2), and six copies of *PML* (15q24) in 34% (68/200) of nuclei, confirming that the chromosome 15 abnormalities involved complex intrachromosomal events rather than simple whole-chromosome gain (Figure 2G).

The ISCN nomenclature of this specimen was established to be 48,X,-Y,der(1)(1pter->1q42::5p15->5pter),+5,-8,der(8)(5pter->5p15::8q11.1->8q24::5q31->5qter),+der(9)(pter->q13::1q21->qter),-13,+15,der(18)(18pter->18q21::15q11.2->15qter),+add(19)(q13.3),+21,der(21)(15qter->15q11.2::21p13->qter)x2,+1mar[cp9].

This particular pattern, featuring gain of 1q, monosomy 13, and extensive copy number alterations of chromosomes 5, 9, and 15 within the context of additional numerical and structural chromosomal abnormalities (-8, -Y, +21, +1mar), appeared suggestive of a chromothripsis-like pattern and indicated high-risk disease according to mSMART 4.0 criteria [25,26].

### 2.5. Treatment and Response

The patient underwent three sessions of plasma exchange (PLEX) to treat presumed myeloma cast nephropathy. Shortly after the first PLEX session, chemotherapy was initiated with cyclophosphamide, bortezomib, and dexamethasone (CyBorD) combined with the monoclonal antibody daratumumab.

After one cycle of CyBorD plus daratumumab, the patient achieved a dramatic hematologic response, with serum-free lambda decreasing to 6.48 mg/L (>99% reduction from initial 3850 mg/L) and serum IgG decreasing to 715 mg/dL. Remarkably, complete renal recovery was achieved, with creatinine normalizing to 0.86 mg/dL and normalization of electrolytes, allowing for permanent discontinuation of hemodialysis at one week post-discharge.

The patient subsequently tolerated and completed four 28-day cycles of CyBorD plus daratumumab induction. A follow-up bone marrow biopsy performed three months after the initial diagnosis demonstrated a normocellular marrow with no morphologic or flow cytometric evidence of residual plasma cell myeloma. Importantly, repeat FISH analysis was negative for all previously identified abnormalities, indicating complete cytogenetic remission. The patient is currently undergoing preparation for consolidation therapy with an autologous stem cell transplant.

## 3. Discussion

This case represents a remarkable clinical scenario of high-risk PCM with complex cytogenetic abnormalities achieving excellent therapeutic response to quadruplet therapy. The cytogenetic findings and clinical implications warrant detailed discussion and literature review.

### 3.1. Complex Cytogenetic Profile Indicating High-Risk Stratification

Comprehensive FISH analysis revealed a complex pattern of numerical chromosomal abnormalities affecting multiple chromosomes. The presence of 1q gain at high clonal fraction is particularly significant, indicating that this may represent a founder event in this patient. Recent studies suggest that early acquisition of 1q gain is associated with outcomes comparable to 1q amplification (>3 copies) [27,29,30].

The detection of monosomy 13 further compounds the adverse prognosis. While hemizygous deletion of 13q is common in PCM, complete monosomy 13 has been linked to the transition from MGUS to PCM and correlates with significantly inferior outcomes, particularly in the context of other high-risk features [28,31,32].

Most notably, the chromosome 15 abnormalities reveal a distinct “shattered” signature. An unusual and complex pattern of intrachromosomal gain was detected, revealing variable amplifications of the *SNRPN* (15q11.2) and *PML* (15q24) loci. The discordance between centromeric and locus-specific copy numbers strongly implies chromothripsis-like rearrangement with subsequent amplification of specific segments [17].

Collectively, this constellation of 1q gain, monosomy 13, and complex structural rearrangements of chromosomes 5, 9, and 15 indicates a high degree of genomic instability and potentially aggressive disease biology [18,19,27]. Based on both the mSMART 4.0 and the IMS-IMWG CGS risk criteria for PCM, this patient would be definitively classified as high-risk due to the presence of 1q gain with additional cytogenetic abnormalities [25,26,28]. Critically, the presence of multiple high-risk cytogenetic abnormalities (often termed “double-hit” or “triple-hit” PCM) is associated with cumulative adverse effects on survival and rapid development of treatment resistance, suggesting that the increased degree of genomic instability seen in chromothripsis-like patterns may exceed current high-risk stratification [22,23].

### 3.2. Exceptional Response Despite High-Risk Cytogenetics

Despite this high-risk cytogenetic profile, the patient achieved a dramatic hematologic response after one cycle of CyBorD plus daratumumab, with serum-free lambda decreasing by >99% and complete renal recovery. This response is particularly noteworthy given that patients with 1q gain often achieve good initial response but may suffer from early relapse and inferior long-term survival [27].

The addition of daratumumab to bortezomib-based therapy likely made it possible to overcome the adverse cytogenetic profile. While high-risk genomic features, such as chromothripsis and 1q gain, typically confer drug resistance by driving rapid cellular proliferation and impairing DNA damage repair pathways, the immune-mediated cytotoxicity of CD38-targeting daratumumab likely functions independently of intracellular defects, which may allow for effective bypass of this intrinsic resistance driven by the genomic profile [33]. Recent trials have demonstrated that intensification with monoclonal antibody-based quadruplet regimens may partially mitigate the poor prognosis associated with high-risk cytogenetics [34,35]. Furthermore, bortezomib-based regimens have shown particular efficacy in cast nephropathy regardless of cytogenetic risk; the rapid and complete renal recovery in this case aligns with recent data showing that effective myeloma therapy may reverse renal failure in over 50% of dialysis-dependent patients [36,37,38].

### 3.3. Prognostic Implications and Long-Term Management

Given the high-risk cytogenetic profile with features suggestive of chromothripsis, this patient requires intensive longitudinal monitoring for relapse despite excellent initial response. Historical data suggest that patients with chromothripsis face a dismal prognosis associated with rapid disease progression and significantly inferior survival compared to standard-risk patients [17,20,22]. Moreover, those with combined 1q gain and monosomy 13 have significantly worse outcomes than those with either abnormality alone [27,28,32]. However, the dramatic response to quadruplet therapy including daratumumab suggests a therapeutic vulnerability that could be exploited with continued aggressive treatment and maintenance strategies [34]. Indeed, to minimize the high risk of early relapse associated with this genomic profile, the patient’s initial therapeutic response (confirmed via morphology, flow cytometry, and cytogenetics) is currently being consolidated with autologous stem cell transplantation. While the durability of this response remains to be fully seen, the recent advent of novel T-cell redirecting immunotherapies, such as bispecific antibodies and chimeric antigen receptor T-cell therapy, provides promising future salvage strategies should this high-risk biology eventually drive disease recurrence [39,40]. Consequently, identifying specific prognostic features is crucial for selecting patients who may benefit from risk-adapted treatment intensification, novel therapeutic approaches, or clinical trial enrollment [3,22].

### 3.4. Technical Considerations

This case demonstrates the value of comprehensive FISH panel design and the use of CD138-enriched plasma cells for optimal detection of cytogenetic abnormalities [2,41]. The use of multiple probe sets targeting different chromosomal regions allowed for detailed characterization of the complex chromosomal abnormalities. According to recent guidelines from the Cancer Genomics Consortium Plasma Cell Neoplasm Working Group, standardization in FISH testing and reporting practices is essential to improve report clarity and align with updated risk stratification systems [41]. Accurate application of ISCN nomenclature was essential to reflect all detected abnormalities, including the complex pattern on chromosome 15 and the copy number abnormalities suggestive of a chromothripsis-like pattern [2,42]. We acknowledge that while our FISH and karyotype data strongly suggest this phenomenon, the definitive gold standard for diagnosing true chromothripsis requires whole-genome sequencing, which may not be routinely available in standard clinical workflows [19,20]. Therefore, these cytogenetic findings would be most appropriately classified as a chromothripsis-like genomic profile. Finally, proper documentation of the percentage of cells harboring each abnormality is critical for assessing clonal architecture and potential subclonal populations, as these features may drive disease evolution and therapeutic resistance [2,41].

## 4. Conclusions

This case highlights that robust clinical responses are achievable in high-risk PCM using contemporary quadruplet regimens, even in the setting of complex genomic instability. The observed pattern of chromosomal abnormalities, including gain of 1q, monosomy 13, and chromothripsis-like structural variants, represents a high-risk cytogenetic profile typically associated with poor outcomes. Yet, the rapid hematologic and renal recovery following CyBorD plus daratumumab, culminating in complete cytogenetic remission after four cycles, challenges the expectation of early treatment failure in this population. While this deep initial response successfully bridged the patient to consolidation via autologous stem cell transplantation, the long-term durability of such responses in high-risk, genomically unstable PCM cases warrants ongoing study. Given that patients with this degree of genomic instability remain prone to clonal evolution and relapse, ongoing comprehensive cytogenetic monitoring will be critical. Ultimately, this case underscores the critical importance of utilizing comprehensive FISH panels to identify high-risk PCM biology at diagnosis and to guide risk-adapted therapeutic strategies.

## Data Availability

The original contributions presented in this study are included in the article. Further inquiries can be directed to the corresponding author.
